# A general-purpose material property data extraction pipeline from large polymer corpora using natural language processing

**DOI:** 10.1038/s41524-023-01003-w

**Published:** 2023-04-05

**Authors:** Pranav Shetty, Arunkumar Chitteth Rajan, Chris Kuenneth, Sonakshi Gupta, Lakshmi Prerana Panchumarti, Lauren Holm, Chao Zhang, Rampi Ramprasad

**Affiliations:** 1 School of Computational Science & Engineering, Atlanta, GA USA; 2 School of Materials Science and Engineering, Georgia Institute of Technology, 771 Ferst Drive NW, Atlanta, 30332 GA USA; 3 Department of Metallurgy Engineering and Materials Science, Indian Institute of Technology, Indore, Madhya Pradesh India

**Keywords:** Computational methods, Polymers

## Abstract

The ever-increasing number of materials science articles makes it hard to infer chemistry-structure-property relations from literature. We used natural language processing methods to automatically extract material property data from the abstracts of polymer literature. As a component of our pipeline, we trained MaterialsBERT, a language model, using 2.4 million materials science abstracts, which outperforms other baseline models in three out of five named entity recognition datasets. Using this pipeline, we obtained ~300,000 material property records from ~130,000 abstracts in 60 hours. The extracted data was analyzed for a diverse range of applications such as fuel cells, supercapacitors, and polymer solar cells to recover non-trivial insights. The data extracted through our pipeline is made available at polymerscholar.org which can be used to locate material property data recorded in abstracts. This work demonstrates the feasibility of an automatic pipeline that starts from published literature and ends with extracted material property information.

## Introduction

The number of materials science papers published annually grows at the rate of 6% compounded annually. Quantitative and qualitative material property information is locked away in these publications written in natural language that is not machine-readable. The explosive growth in published literature makes it harder to see quantitative trends by manually analyzing large amounts of literature. Searching the literature for material systems that have desirable properties also becomes more challenging. Moreover, material information published in a non-machine-readable form contributes to data scarcity in the field of materials informatics where the training of property predictors requires painstaking manual curation of the data of interest from literature. Here, we propose adapting techniques for information extraction from the natural language processing (NLP) literature to address these issues.

Information extraction from the written text is well-studied within NLP and involves several key components such as named entity recognition (NER), i.e., identifying categories to which words in the text belong; relation extraction, i.e., classifying relationships between extracted entities; co-referencing, i.e., identifying clusters of named entities in the text referring to the same object such as a polymer and its abbreviation, and named entity normalization, i.e., identifying all the variations in the name for an entity across a large number of documents. The idea of “self-supervised learning” through transformer-based models such as BERT^1,2^, pre-trained on massive corpora of unlabeled text to learn contextual embeddings, is the dominant paradigm of information extraction today. A common architecture for NER and relation extraction is to feed a labeled input to BERT and use the output vector embedding for each word along with the corresponding labels (which could be entity labels or relation labels) as inputs to a task-specific machine learning model (typically a neural network) that learns to predict those labels. The tasks mentioned above are label intensive. Extending these methods to new domains requires labeling new data sets with ontologies that are tailored to the domain of interest.

ChemDataExtractor^3^, ChemSpot^4^, and ChemicalTagger^5^ are tools that perform NER to tag material entities. For example, ChemDataExtractor has been used to create a database of Neel temperatures and Curie temperatures that were automatically mined from literature^6^. It has also been used to generate a literature-extracted database of magnetocaloric materials and train property prediction models for key figures of merit^7^. In the space of polymers, the authors of Ref. ^8^ used a semi-automated approach that crawled papers automatically and used students to extract the Flory-Huggins parameter (a measure of the affinity between two materials, eg., a polymer and a solvent). Word embedding approaches were used in Ref. ^9^ to generate entity-rich documents for human experts to annotate which were then used to train a polymer named entity tagger. Most previous NLP-based efforts in materials science have focused on inorganic materials^10,11^ and organic small molecules^12,13^ but limited work has been done to address information extraction challenges in polymers. Polymers in practice have several non-trivial variations in name for the same material entity which requires polymer names to be normalized. Moreover, polymer names cannot typically be converted to SMILES strings^14^ that are usable for training property-predictor machine learning models. The SMILES strings must instead be inferred from figures in the paper that contain the corresponding structure.

Past work to automatically extract material property information from literature has focused on specific properties typically using keyword search methods or regular expressions^15^. However, there are few solutions in the literature that address building general-purpose capabilities for extracting material property information, i.e., for any material property. Moreover, property extraction and analysis of polymers from a large corpus of literature have also not yet been addressed. Automatically analyzing large materials science corpora has enabled many novel discoveries in recent years such as Ref. ^16^, where a literature-extracted data set of zeolites was used to analyze interzeolite relations. Using word embeddings trained on such corpora has also been used to predict novel materials for certain applications in inorganics and polymers^17,18^.

**Fig. 1:**
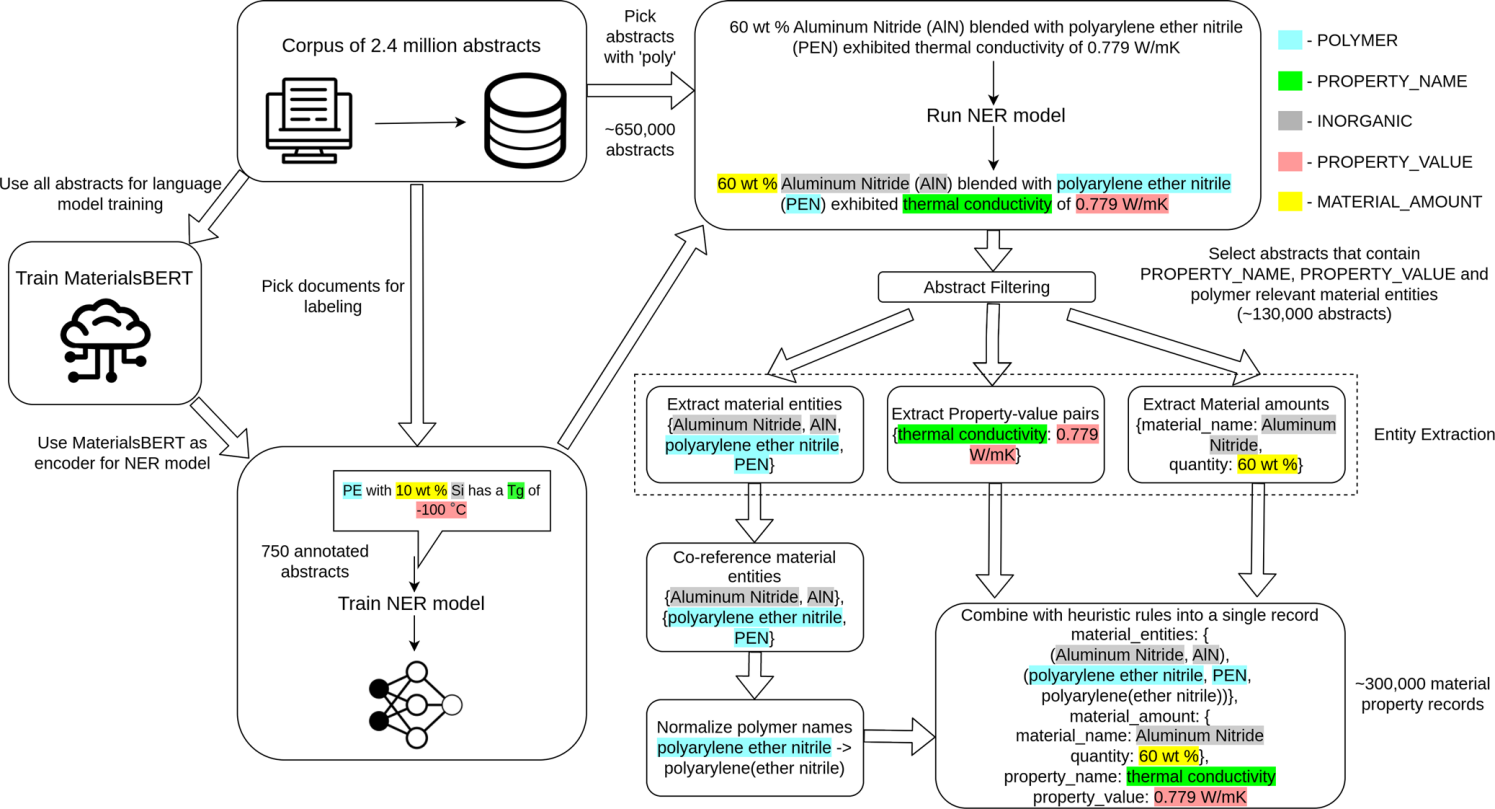
Pipeline used for extracting material property records from a corpus of abstracts. The training of MaterialsBERT, training of the NER model as well as the use of the NER model in conjunction with heuristic rules to extract material property data.

We built a general-purpose pipeline for extracting material property data in this work. Starting with a corpus of 2.4 million materials science papers described in Ref. ^17^, we selected 750 abstracts from the polymer domain and annotated each of the abstracts using our own ontology that was designed for the purpose of extracting information from materials science literature. Using these 750 annotated abstracts we trained an NER model, using our MaterialsBERT language model to encode the input text into vector representations. MaterialsBERT in turn was trained by starting from PubMedBERT, another language model, and using 2.4 million materials science abstracts to continue training the model^19^. The trained NER model was applied to polymer abstracts and heuristic rules were used to combine the predictions of the NER model and obtain material property records from all polymer-relevant abstracts. This pipeline is illustrated in Fig. 1. We restricted our focus to abstracts as associating property value pairs with their corresponding materials is a more tractable problem in abstracts. We analyzed the data obtained using this pipeline for applications as diverse as polymer solar cells, fuel cells, and supercapacitors and showed that several known trends and phenomena in materials science can be inferred using this data. Moreover, we trained a machine learning predictor for the glass transition temperature using automatically extracted data (Supplementary Discussion 3).

This work builds a general-purpose material property data extraction pipeline, for any material property. MaterialsBERT, the language model that powers our information extraction pipeline is released in order to enable the information extraction efforts of other materials researchers. There are other BERT-based language models for the materials science domain such as MatSciBERT^20^ and the similarly named MaterialBERT^21^ which have been benchmarked on materials science specific NLP tasks. This work goes beyond benchmarking the language model on NLP tasks and demonstrates how it can be used in combination with NER and relation extraction methods to extract all material property records in the abstracts of our corpus of papers. In addition, we show that MaterialsBERT outperforms other similar BERT-based language models such as BioBERT^22^ and ChemBERT^23^ on three out of five materials science NER data sets. The data extracted using this pipeline can be explored using a convenient web-based interface (polymerscholar.org) which can aid polymer researchers in locating material property information of interest to them.

## Results

### Abstract annotation

Our ontology for extracting material property information consists of 8 entity types namely POLYMER, POLYMER_CLASS, PROPERTY_VALUE, PROPERTY_NAME, MONOMER, ORGANIC_MATERIAL, INORGANIC_MATERIAL, and MATERIAL_AMOUNT. For a detailed description of these entity types, see Table 1. This ontology captures the key pieces of information commonly found in abstracts and the information we wish to utilize for downstream purposes. Unlike some other studies^24^, our ontology does not annotate entities using the BIO tagging scheme, i.e., **B**eginning-**I**nside-**O**utside of the labeled entity. Instead, we opt to keep the labels simple and annotate only tokens belonging to our ontology and label all other tokens as ‘OTHER’. This is because, as reported in Ref. ^19^, for BERT-based sequence labeling models, the advantage offered by explicit BIO tags is negligible and IO tagging schemes suffice. More detailed annotation guidelines are provided in Supplementary Methods 1. The corpus of papers described previously was filtered to obtain a data set of abstracts that were polymer relevant and likely to contain the entity types of interest to us. We did so by filtering abstracts containing the string ‘poly’ to find polymer-relevant abstracts and using regular expressions to find abstracts that contained numeric information.

**Table 1:** Description of each entity type in the ontology used for annotating PolymerAbstracts.

Entity type	Description	Total occurrences
POLYMER	Material entities that are polymers	7364
ORGANIC_MATERIAL	Material entities that are organic but not polymers. Typically used as plasticizers or cross-linking agents	914
MONOMER	Material entities which are explicitly indicated as being the repeat units for a POLYMER entity	2074
POLYMER_CLASS	Material entities that don’t refer to a specific chemical substance but are broad terms used for a class of polymers	1476
INORGANIC_MATERIAL	Material entities which are inorganic and are typically used as additives in a polymer formulation	1272
MATERIAL_AMOUNT	Entity type indicating the amount of a particular material in a material formulation	1143
PROPERTY_NAME	Entity type for a material property	4535
PROPERTY_VALUE	Entity type including a numeric value and its unit corresponding to a material property	5800
OTHER	Default entity type used for all tokens that do not lie in any of the above categories	147,115

Total occurrences here refers to the number of occurrences of each entity type in PolymerAbstracts.

Using the above-described ontology, we annotated 750 abstracts and split the abstracts into 85% for training, 5% for validation, and 10% for testing. Prior to manual annotation, we automatically pre-annotated the abstracts using dictionaries of entities for the entity types where one was available^24^. This was intended to speed up the annotation process. This data set was annotated by three domain experts using the tool Prodigy (https://prodi.gy). Annotation was done over three rounds using a small sample of abstracts in each round. With each round, the annotation guidelines were refined and the abstracts in the previous rounds were re-annotated using the refined guidelines. We refer to this data set as PolymerAbstracts.

In order to assess the inter-annotator agreement between the three annotators, 10 abstracts were annotated by all the annotators to measure the Cohen’s Kappa and Fleiss Kappa^25^ metrics. The Fleiss Kappa metric was computed to be 0.885 and the pairwise Cohen’s Kappa metric to be (0.906, 0.864, 0.887) for each of the three pairs of annotators. These metrics are comparable to inter-annotator agreements reported elsewhere in the literature^26^ and indicate good homogeneity in the annotations.

### NER model

**Fig. 2:**
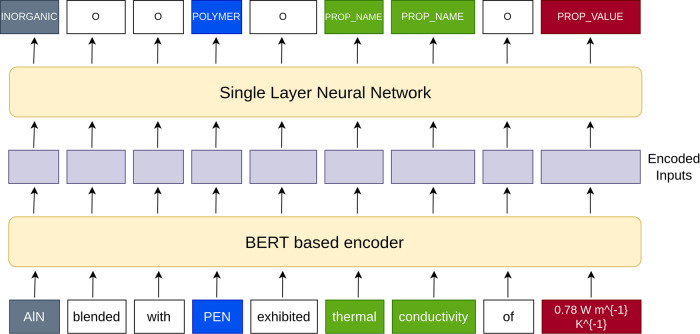
Model architecture used for named entity recognition. Each token in the input sequence is converted to a contextual embedding by a BERT-based encoder which is then input to a single-layer neural network. The output of the neural network is the entity type of the input token.

The architecture used for training our NER model is depicted in Fig. 2. BERT and BERT-based models have become the de-facto solutions for a large number of NLP tasks^1^. It embodies the transfer learning paradigm in which a language model is trained on a large amount of unlabeled text using unsupervised objectives (not shown in Fig. 2) and then reused for other NLP tasks. The resulting BERT encoder can be used to generate token embeddings for the input text that are conditioned on all other input tokens and hence are context-aware. We used a BERT-based encoder to generate representations for tokens in the input text as shown in Fig. 2. The generated representations were used as inputs to a linear layer connected to a softmax non-linearity that predicted the probability of the entity type of each token. The cross-entropy loss was used during training to learn the entity types and on the test set, the highest probability label was taken to be the predicted entity type for a given input token. Dropout was used in the linear layer with a dropout probability of 0.2. The BERT model has an input sequence length limit of 512 tokens and most abstracts fall within this limit. Sequences longer than this length were truncated to 512 tokens as per standard practice^27^. We used a number of different encoders and compared the performance of the resulting models on PolymerAbstracts. We compared these models for a number of different publicly available materials science data sets as well. All experiments were performed by us and the training and evaluation setting was identical across the encoders tested, for each data set.

### Evaluation methods

The performance of the NER model is evaluated using precision, recall, and F1 score of the predicted entity tag compared to the ground truth labels. These are defined below:

1 $$\begin{array}{l}{{{\rm{Precision}}}}\,=\,\frac{{{{\rm{TP}}}}}{{{{\rm{TP}}}}\,+\,{{{\rm{FP}}}}}\\ {{{\rm{Recall}}}}\,=\,\frac{{{{\rm{TP}}}}}{{{{\rm{TP}}}}\,+\,{{{\rm{FN}}}}}\\ {{{\rm{F1}}}}\,=\,\frac{2\times {{{\rm{Precision}}}}\times {{{\rm{Recall}}}}}{{{{\rm{Precision}}}}\,+\,{{{\rm{Recall}}}}}\end{array}$$

where TP are the true positives, FP are the false positives and FN are the false negatives. Each of the above metrics is reported as a % value. We consider a predicted label to be a true positive only when the label of a complete entity is predicted correctly. For instance, for the polymer ‘polyvinyl ethylene’, both ‘polyvinyl’ and ‘ethylene’ must be correctly labeled as a POLYMER, else the entity is deemed to be predicted incorrectly.

### NER model performance

The performance of various BERT-based language models tested for training an NER model on PolymerAbstracts is shown in Table 2. We observe that MaterialsBERT, the model fine-tuned by us on 2.4 million materials science abstracts using PubMedBERT as the starting point, outperforms PubMedBERT as well as other language models used. This is in agreement with previously reported results where the fine-tuning of a BERT-based language model on a domain-specific corpus resulted in improved downstream task performance^19^. Similar trends are observed across two of the four materials science data sets as reported in Table 3 and thus MaterialsBERT outperforms other BERT-based language models in three out of five materials science data sets. These NER datasets were chosen to span a range of subdomains within materials science, i.e., across organic and inorganic materials. A more detailed description of these NER datasets is provided in Supplementary Methods 2. All encoders tested in Table 2 used the BERT-base architecture, differing in the value of their weights but having the same number of parameters and hence are comparable. MaterialsBERT outperforms PubMedBERT on all datasets except ChemDNER, which demonstrates that fine-tuning on a domain-specific corpus indeed produces a performance improvement on sequence labeling tasks. ChemBERT^23^ is BERT-base fine-tuned on a corpus of ~400,000 organic chemistry papers and also out-performs BERT-base^1^ across the NER data sets tested. BioBERT^22^ was trained by fine-tuning BERT-base using the PubMed corpus and thus has the same vocabulary as BERT-base in contrast to PubMedBERT which has a vocabulary specific to the biomedical domain. Ref. ^28^ describes the model MatBERT which was pre-trained from scratch using a corpus of 2 million materials science articles. Despite MatBERT being a model that was pre-trained from scratch, MaterialsBERT outperforms MatBERT on three out of five datasets. While the vocabulary of MatBERT and MaterialsBERT are both relevant to the domain of materials science, this performance difference can likely be attributed to the fact that PubMedBERT, the initial model for MaterialsBERT was pre-trained on a much larger corpus of text (14 million abstracts and full text). All experiments shown in Tables 2 and 3 were performed by us. We did not test BiLSTM-based architectures^29^ as past work has shown that BERT-based architectures typically outperform BiLSTM-based ones^19,23,28^. The performance of MaterialsBERT for each entity type in our ontology is described in Supplementary Discussion 1.

**Table 2:** Performance of various BERT-based encoders on the test set of PolymerAbstracts.

Model	Precision	Recall	F1
MaterialsBERT (ours)	62.5	70.6	**66.4**
PubMedBERT	61.4	70.7	65.8
MatBERT	60.9	70.1	65.2
BioBERT	59.2	66.3	62.6
ChemBERT	52.2	62.6	57.0
BERT-base	52.1	61.0	56.2

Values are reported in %.

MaterialsBERT has the highest F1 score (shown in bold).

**Table 3:** Performance of different BERT-based encoders on the test sets of publicly available materials science NER datasets.

BERT-based encoder	ChemDNER^70^			Inorganic Synthesis recipes^71^			Inorganic Abstracts^24^			ChemRxnExtractor^23^		
	P	R	F1	P	R	F1	P	R	F1	P	R	F1
MaterialsBERT (ours)	70.1	68.2	69.2	69.1	68.3	**68.6**	85.3	86.7	86.0	73.5	69.5	**71.4**
PubMedBERT	71.5	69.0	**70.2**	69.9	65.3	67.6	84.0	86.2	85.0	68.1	59.5	63.6
MatBERT	71.7	66.9	69.2	68.6	67.7	68.2	85.6	86.7	86.2	67.4	58.0	62.4
BioBERT	70.6	65.7	68.0	64.4	63.7	64.0	85.6	87.1	**86.4**	74.8	65.4	69.8
ChemBERT	72.5	66.4	69.4	66.8	64.3	65.6	83.2	86.4	84.8	65.0	64.0	64.4
BERT-base	71.2	65.7	68.4	62.4	60.3	61.4	81.0	81.9	81.4	57.7	54.9	56.2

Values are reported in %.

The encoders with the highest F1 score for each dataset tested are shown in bold.

### Quantifying the extracted data

Using our pipeline, we extracted ~300,000 material property records from ~130,000 abstracts. Out of our corpus of 2.4 million articles, ~650,000 abstracts are polymer relevant and around ~130,000 out of those contain material property data. This extraction process took 60 hours using a single Quadro 16 GB GPU. To place this number in context, PoLyInfo a comparable database of polymer property records that is publicly available has 492,645 property records as of this writing^30^. This database was manually curated by domain experts over many years while the material property records we have extracted using automated methods took 2.5 days using only abstracts and is yet of comparable size. However, the curation of datasets is not eliminated by automated extraction as we will still need domain experts to carefully curate text-mined data sets but these methods can dramatically reduce the amount of work needed. It is easier to flag bad entries in a structured format than to manually parse and enter data from natural language. The composition of these material property records is summarized in Table 4 for specific properties (grouped into a few property classes) that are utilized later in this paper. For the general property class, we computed the number of neat polymers as the material property records corresponding to a single material of the POLYMER entity type. Blends correspond to material property records with multiple POLYMER entities while composites contain at least one material entity that is not of the POLYMER or POLYMER_CLASS entity type. To compute the number of unique neat polymer records, we first counted all unique normalized polymer names from records that had a normalized polymer name. This accounts for the majority of polymers with multiple reported names as detailed in Ref. ^31^. Out of the remaining neat polymer records that did not have a normalized polymer name, we then counted all unique polymer names (accounting for case variations) and added them to the number of unique normalized polymer names to arrive at the estimated number of unique polymers. For the general property class, we note that elongation at break data for an estimated 413 unique neat polymers was extracted. In contrast, Ref. ^32^ used 77 polymers to train a machine learning model. For tensile strength, an estimated 926 unique neat polymer data points were extracted while Ref. ^33^ used 672 data points to train a machine learning model. Thus the amount of data extracted in the aforementioned cases by our pipeline is already comparable to or greater than the amount of data being utilized to train property predictors in the literature. Table 4 accounts for only 39207 data points which is 13% of the total extracted material property records. More details on the extracted material property records can be found in Supplementary Discussion 2. The reader is also encouraged to explore this data further through polymerscholar.org.

**Table 4:** Number of material property records extracted for several key polymer properties and figures of merit for certain applications.

Property class	Property	Total number of datapoints	neat polymers/ blends/ composites	Estimated number of unique neat polymers
General	Molecular Weight	9053	9053/-/-	2623
	Glass Transition Temperature	6155	4612/1036/507	1732
	Electrical conductivity	6030	3202/606/2222	1017
	Tensile Strength	4382	2679/651/1052	926
	Elongation at Break	1499	954/234/311	413
Polymer Solar Cells	Power Conversion Efficiency	3595	–	–
	Open Circuit Voltage	1386	–	–
	Short Circuit Current	1049	–	–
	Fill Factor	966	–	–
Fuel Cells	Proton conductivity	1359	–	–
	Areal Power Density	1235	–	–
	Areal Current Density	295	–	–
	Methanol permeability	174	–	–
Supercapacitors	Gravimetric Energy Density	1131	–	–
	Gravimetric Power Density	898	–	–

### General property class

**Fig. 3:**
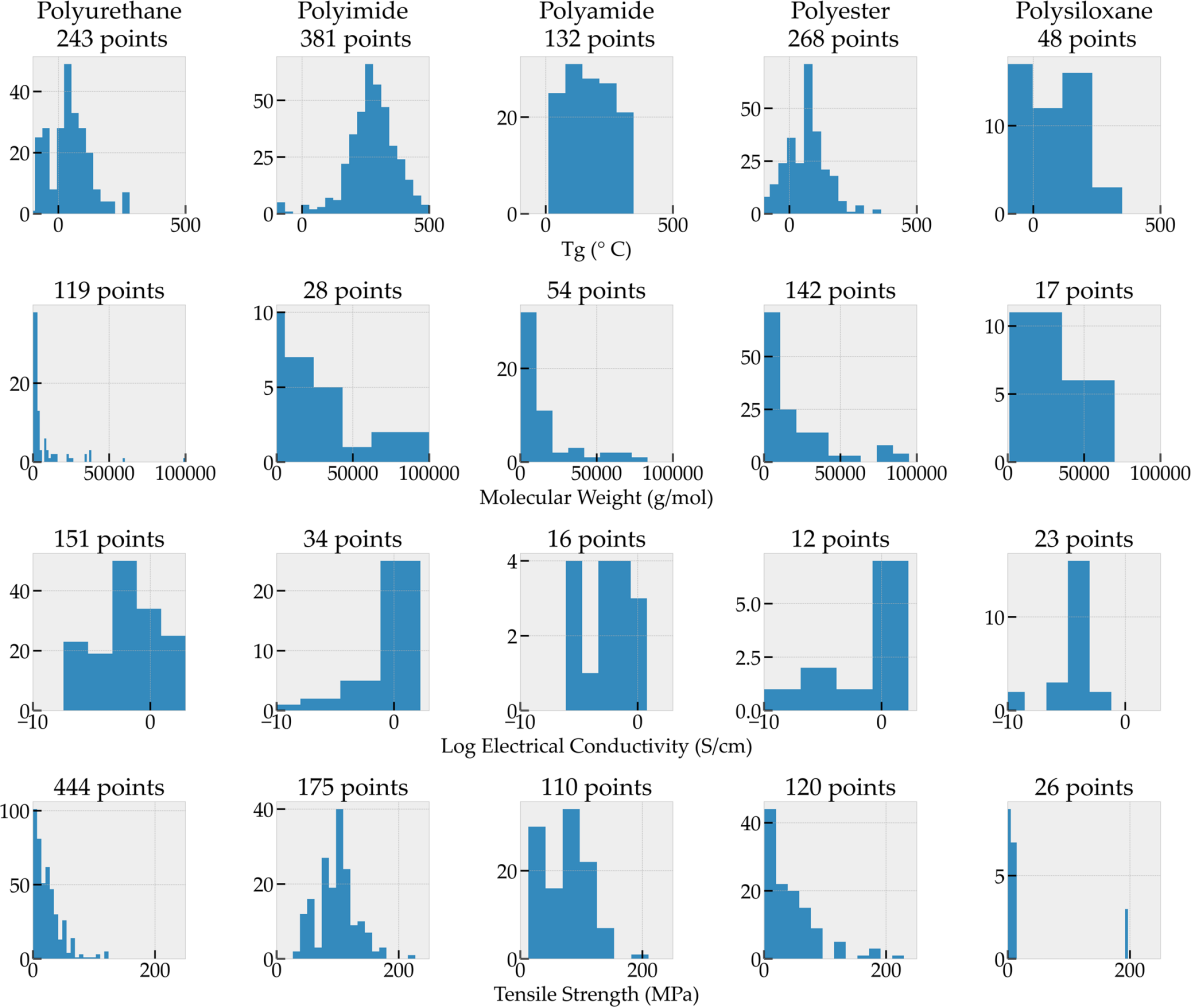
Material property data extracted from abstracts for material systems that contain a polymer from the polymer classes of polyurethane, polyimide, polyamide, polyester, and polysiloxane in each corresponding column. These are the most commonly reported polymer classes and the properties reported are the most commonly reported properties in our corpus of papers.

We now analyze the properties extracted class-by-class in order to study their qualitative trend. Figure 3 shows property data extracted for the five most common polymer classes in our corpus (columns) and the four most commonly reported properties (rows). Polymer classes are groups of polymers that share certain chemical attributes such as functional groups. The properties reported in Fig. 3 fall under the general property class described in Table 4. The material property data in Fig. 3 corresponds to cases when a polymer of a particular polymer class is part of the formulation for which a property is reported and does not necessarily correspond to homopolymers but instead could correspond to blends or composites. The polymer class is “inferred” through the POLYMER_CLASS entity type in our ontology and hence must be mentioned explicitly for the material property record to be part of this plot. Several key trends are captured in this plot. From the glass transition temperature (*T*_g_) row, we observe that polyamides and polyimides typically have higher *T*_g_ than other polymer classes. Molecular weights unlike the other properties reported are not intrinsic material properties but are determined by processing parameters. The reported molecular weights are far more frequent at lower molecular weights than at higher molecular weights; mimicking a power-law distribution rather than a Gaussian distribution. This is consistent with longer chains being more difficult to synthesize than shorter chains. For electrical conductivity, we find that polyimides have much lower reported values which is consistent with them being widely used as electrical insulators. Also note that polyimides have higher tensile strengths as compared to other polymer classes, which is a well-known property of polyimides^34^.

**Fig. 4:**
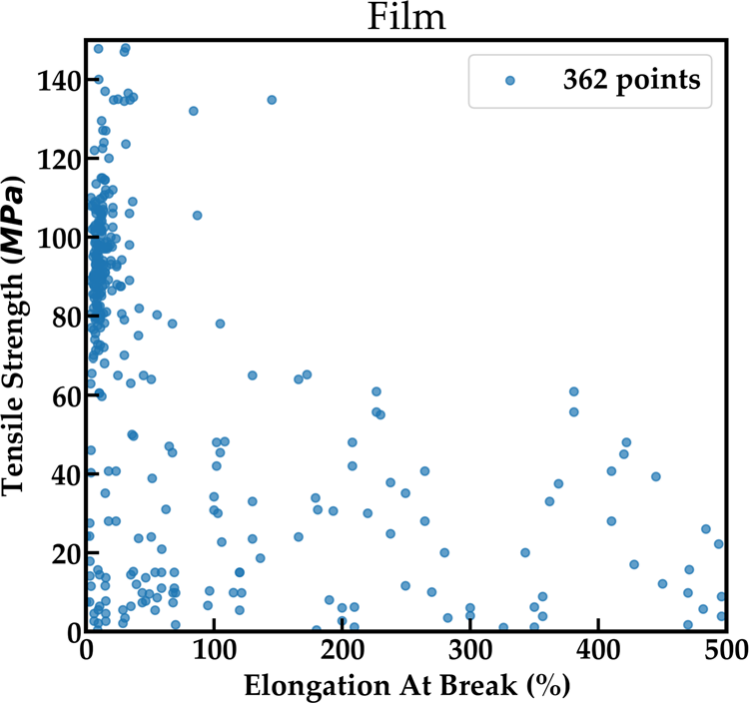
Tensile Strength Vs Elongation at break for films demonstrating the strength-ductility trade-off. Materials with high elongation at break demonstrate lower tensile strength and conversely, those with high tensile strength have lower elongation at break.

Figure 4 shows mechanical properties measured for films which demonstrates the trade-off between elongation at break and tensile strength that is well known for materials systems (often called the strength-ductility trade-off dilemma). Materials with high tensile strength tend to have a low elongation at break and conversely, materials with high elongation at break tend to have low tensile strength^35^. This known fact about the physics of material systems emerges from an amalgamation of data points independently gathered from different papers. In the next section, we take a closer look at pairs of properties for various devices that reveal similarly interesting trends.

### Knowledge extraction

**Fig. 5:**
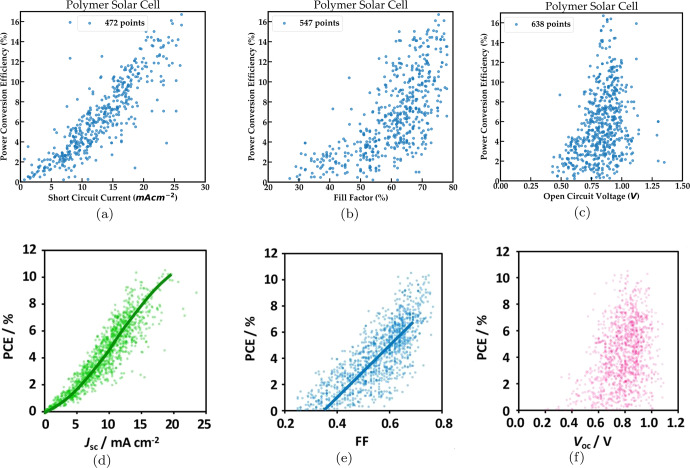
Correlations between key properties extracted automatically from literature for polymer solar cells. **a** Power conversion efficiency against short circuit current **b** Power conversion efficiency against fill factor **c** Power conversion efficiency against open circuit voltage. Correlations between key properties extracted manually from literature for polymer solar cells **d** Power conversion efficiency against short circuit current **e** Power conversion efficiency against fill factor **f** Power conversion efficiency against open circuit voltage. Figure 5**d**–**f** is adapted here with permission from Ref. ^37^. Copyright 2018 American Chemical Society.

**Fig. 6:**
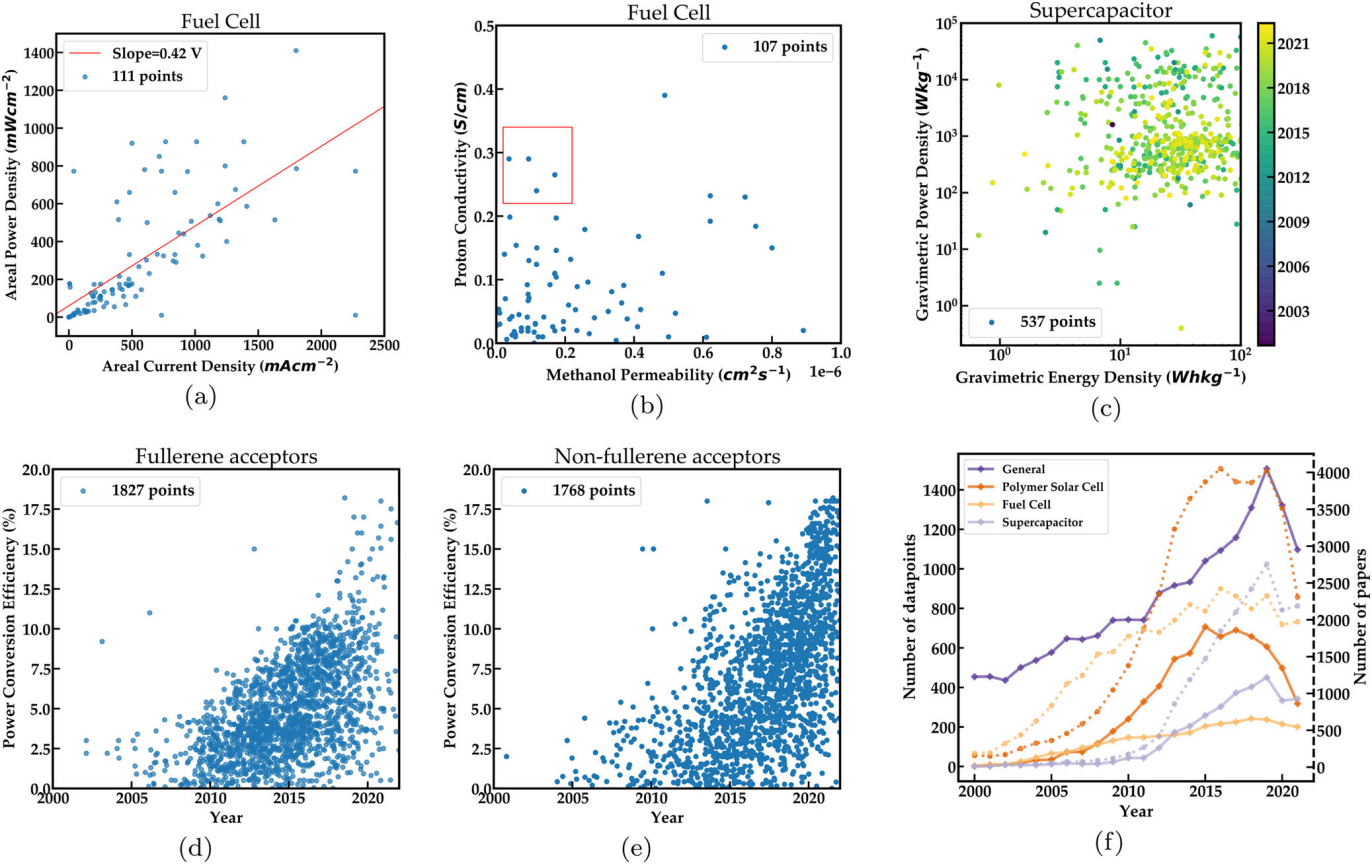
Correlations between key properties extracted automatically from literature for three different applications. **a** Areal current density Vs Areal power density for fuel cells. the slope of the best-fit line has a slope of 0.42 V which is the typical operating voltage of a fuel cell **b** Proton conductivity vs. Methanol permeability for fuel cells. The red box shows the desirable region of the property space **c** Up-to-date Ragone plot for supercapacitors showing energy density Vs power density. **d** lower conversion efficiency against time for fullerene acceptors and **e** Power conversion efficiency against time for non-fullerene acceptors **f** Trend of the number of data points extracted by our pipeline over time. The dashed lines represent the number of papers published for each of the three applications in the plot and correspond to the dashed Y-axis.

Next, we consider a few device applications and co-relations between the most important properties reported for these applications to demonstrate that non-trivial insights can be obtained by analyzing this data. We consider three device classes namely polymer solar cells, fuel cells, and supercapacitors, and show that their known physics is being reproduced by NLP-extracted data. We find documents specific to these applications by looking for relevant keywords in the abstract such as ‘polymer solar cell’ or ‘fuel cell’. The total number of data points for key figures of merit for each of these applications is given in Table 4. The number of extracted data points reported in Table 4 is higher than that in Fig. 5 and Fig. 6 as additional constraints are imposed in the latter cases to better study this data.

#### Polymer solar cells

Polymer solar cells, in contrast to conventional silicon-based solar cells, have the benefit of lower processing costs but suffer from lower power conversion efficiencies. Improving their power conversion efficiency by varying the materials used in the active layer of the cell is an active area of research^36^. Figure 5a–c shows the power conversion efficiency for polymer solar cells plotted against the corresponding short circuit current, fill factor, and open circuit voltage for NLP extracted data while Fig. 5d–f shows the same pairs of properties for data extracted manually as reported in Ref. ^37^. Each data point in Fig. 5a–c is taken from a particular paper and corresponds to a single material system. It is clear from Fig. 5c that the peak power conversion efficiencies reported are around 16.71% which is close to the maximum known values reported in the literature^38^ as of this writing. The open-circuit voltages (OCV) appear to be Gaussian distributed at around 0.85 V. Figure 5a) shows a linear trend between short circuit current and power conversion efficiency. It is clear that the trends observed in Fig. 5a–c for NLP extracted data are quite similar to the trends observed from manually curated data in Fig. 5d–f.

#### Fuel cells

Fuel cells are devices that convert a stream of fuel such as methanol or hydrogen and oxygen to electricity. Water is one of the primary by-products of this conversion making this a clean source of energy. A polymer membrane is typically used as a separating membrane between the anode and cathode in fuel cells^39^. Improving the proton conductivity and thermal stability of this membrane to produce fuel cells with higher power density is an active area of research. Figure 6a and b show plots for fuel cells comparing pairs of key performance metrics. The points on the power density versus current density plot (Fig. 6a)) lie along the line with a slope of 0.42 V which is the typical operating voltage of a fuel cell under maximum current densities^40^. Each point in this plot corresponds to a fuel cell system extracted from the literature that typically reports variations in material composition in the polymer membrane. Figure 6b illustrates yet another use-case of this capability, i.e., to find material systems lying in a desirable range of property values for the more specific case of direct methanol fuel cells. For such fuel cell membranes, low methanol permeability is desirable in order to prevent the methanol from crossing the membrane and poisoning the cathode^41^. High proton conductivity is simultaneously desirable. The box shown in the figure illustrates the desirable region and can thus be used to easily locate promising material systems.

#### Trends across time

We show that known trends across time in polymer literature are also reproduced in our extracted data. A Ragone plot illustrates the trade-off between energy and power density for devices. Supercapacitors are a class of devices that have high power density but low energy density. Figure 6c illustrates the trade-off between gravimetric energy density and gravimetric power density for supercapacitors and is effectively an up-to-date version of the Ragone plot for supercapacitors^42^. Historically, in most Ragone plots, the energy density of supercapacitors ranges from 1 to 10 Wh/kg^43^. However, this is no longer true as several recent papers have demonstrated energy densities of up to 100 Wh/kg^44–46^. As seen in Fig. 6c, the majority of points beyond an energy density of 10 Wh/kg are from the previous two years, i.e., 2020 and 2021.

Figure 6d and e show the evolution of the power conversion efficiency of polymer solar cells for fullerene acceptors and non-fullerene acceptors respectively. These are the two major classes of acceptors used in polymer solar cells. An acceptor along with a polymer donor forms the active layer of a bulk heterojunction polymer solar cell. Observe that more papers with fullerene acceptors are found in earlier years with the number dropping in recent years while non-fullerene acceptor-based papers have become more numerous with time. They also exhibit higher power conversion efficiencies than their fullerene counterparts in recent years. This is a known trend within the domain of polymer solar cells reported in Ref. ^47^. It is worth noting that the authors realized this trend by studying the NLP extracted data and then looking for references to corroborate this observation.

Figure 6f shows the number of data points extracted by our pipeline over time for the various categories described in Table 4. Observe that the number of data points of the general category has grown exponentially at the rate of 6% per year. Out of the three applications considered in Fig. 6f, polymer solar cells have historically had the largest number of papers as well as data points, although that appears to be declining over the past few years. Observe that there is a decline in the number of data points as well as the number of papers in 2020 and 2021. This is likely attributable to the COVID-19 pandemic^48^ which appears to have led to a drop in the number of experimental papers published that form the input to our pipeline^49^.

## Discussion

A natural language processing pipeline that extracts material property records from abstracts has been built and demonstrated. This however has some limitations in practice that we describe below:

1. Material property information is multi-modal and can be found in the text, tables, and figures of the paper. Co-referencing material entity mentions across large spans of text and across figures and tables is a challenging problem. In addition to this, relation extraction of material entities and property value pairs occurring across sentences, are challenges that need to be addressed when extending this work from abstracts to full-text.
2. The ontology used in this work consists of the most important entity types found in materials science literature. This makes it easier to combine material and property information using heuristic rules but misses other information about the material property record such as processing conditions, measurement methods, or measurement conditions which in most cases would influence the property value. This work can be extended to include this metadata by extending the ontology to other entities that would influence the measured property values for any given domain by explicitly labeling those entities and training a new NER model. This approach would however be time-intensive. Another approach would be to use a combination of fully supervised and weakly supervised approaches^50–52^. The ontology used in this work can be used to extract entities like material names as well as property names and values through supervised NER. Our ontology is relevant across all sub-domains of materials science and additional information relevant to a specific sub-domain can be obtained through heuristic rules and regular expressions which would be unsupervised.
3. Converting polymer names to a structure (typically a SMILES string^14^) is also a bottleneck to training property predictor models as this must be done manually. While tools have been developed to handle molecule images^53,54^, reliably and robustly converting images of polymer structures typically found in the literature to SMILES strings is an area of future work for the community. The SMILES string so generated can be used to generate a structural fingerprint vector of the polymer which in turn can serve as the input to a machine learning model. Expanding the scope of this pipeline to figures in the paper would allow training property predictor models without any additional curation for converting images to SMILES strings. Training robust property predictors in this manner would in turn allow the continuous and semi-automatic design of new materials, thus addressing a missing link in materials informatics. An example of manually converting polymer names to SMILES strings followed by the training of a property prediction model for glass transition temperature is shown in Supplementary Discussion 3.

The automated extraction of material property records enables researchers to search through literature with greater granularity and find material systems in the property range of interest. It also enables insights to be inferred by analyzing large amounts of literature that would not otherwise be possible. As shown in the section “Knowledge extraction”, a diverse range of applications were analyzed using this pipeline to reveal non-trivial albeit known insights. This work built a general-purpose capability to extract material property records from published literature. ~300,000 material property records were extracted from ~130,000 polymer abstracts using this capability. Through our web interface (polymerscholar.org) the community can conveniently locate material property data published in abstracts. As part of this work, we also train and release MaterialsBERT, a language model that is fine-tuned on 2.4 million materials science abstracts using PubMedBERT as the starting point and obtains the best F1 score across three of five materials science NER data sets tested.

Growing the extracted material property data set further would require extending this capability to the body of the paper. This would require more robust methods to associate the entities extracted using named entity recognition. A few steps also remain in order to utilize the extracted data to produce trained machine learning property prediction models. The biggest bottleneck in the case of organic materials is obtaining SMILES strings for material entities which can then be used to generate structural fingerprints for property predictor machine learning models. There is also a wealth of additional information such as processing conditions or measurement conditions that are not captured in our ontology. Addressing these bottlenecks would enable automatic and continuous updates of materials databases that can seamlessly power property predictor machine learning models^55,56^.

## Methods

### Corpus of papers

We have created a corpus of ~2.4 million journal articles from the materials science domain. These papers were downloaded from the APIs and websites of publishers such as Elsevier, Wiley, Royal Society of Chemistry, American Chemical Society, Springer Nature, Taylor & Francis, and the American Institute of Physics. The corpus used in this work is an expanded version of the corpus described previously in Ref. ^17^. 750 abstracts of this corpus were annotated and used to train an NER model. Furthermore, the trained NER model along with heuristic rules was used to extract material property records from the abstracts of the full corpus.

### Preprocessing of documents

Because the documents in our corpus are HTML formatted, we stripped all HTML tags to parse the plain text. Moreover, we replaced HTML superscripts and subscripts (<sup> and <sub>) with plain text using the LaTeX convention of ^{} and _ {}, respectively. This is important in order to extract units of quantities as well as property values reported in scientific notation. Property values recorded in this notation were converted back to floating-point numbers downstream when the numeric value was to be recovered. We also mapped characters such as spaces or special characters that have multiple Unicode representations but have a similar appearance by creating a custom mapping.

### Tokenization

For tokenization, i.e., breaking up text into units known as tokens which are used for downstream processing, we used wordpiece tokenization which is the standard tokenization scheme used with BERT and BERT-based models^1,57^. For instance ‘The molecular weight of the result ##ant P ##LL ##A - rich polymer was enhanced .’ is what a sentence would look like post-tokenization. The word ‘resultant’ and the polymer ‘PLLA’ have been broken into sub-word tokens. This is necessary in order to tokenize arbitrary text inputs using a fixed-sized vocabulary as a larger vocabulary would increase the size of the model. Starting with a set of characters (alphabets, numbers, etc), certain combinations of characters are iteratively merged and added to the vocabulary till the vocabulary reaches a certain fixed size^58^. The characters to be merged are selected based on combinations that maximize the likelihood of the input text such that the most frequently occurring sequences of text in the corpus are included in the vocabulary. This typically breaks up words into meaningful subunits such as ‘resultant’ being separated into ‘result’ and ‘##ant’ which reduces the size of the vocabulary. This does not always happen though, as seen with the example of ‘PLLA’. The embedding associated with the first subword for each word is used as the input to the NER model in accordance with conventional practice^1,28^. Thus, only the label predicted for the first subword is used for evaluating the model predictions.

### NER model training

We used the Adam optimizer with an initial learning rate of 5 × 10^−5^ which was linearly damped to train the model^59^. We used early stopping while training the NER model, i.e., the number of epochs of training was determined by the peak F1 score of the model on the validation set as evaluated after every epoch of training^60^. During, this stage, also referred to as ‘fine-tuning’ the model, all the weights of the BERT-based encoder and the linear classifier are updated.

### Training MaterialsBERT

BERT-base, the original BERT model, was trained using an unlabeled corpus that included English Wikipedia and the Books Corpus^61^. The training objectives included using the masked language modeling task, which masks a random subset of the input text and asks the language model to predict it, and the next sentence prediction task which determines for a given sentence pair whether one sentence follows the other in the training data^1^. The vocabulary of the tokenizer was fixed at 30,000 tokens. It is known that a domain-specific BERT encoder improves performance on NLP tasks for that domain because the vocabulary used for tokenization is more representative of the application of interest and because the unlabeled text is also closer to the domain of interest resulting in “better" contextual embeddings^19^. BERT-base was pre-trained from scratch using a general English language corpus^22^.

Even though computationally expensive, pre-training NLP models from scratch has the advantage of creating a model with a vocabulary that is customized for the domain of interest. To give an idea of how resource-intensive this can be, note that RoBERTa, a similarly pre-trained encoder used the computing power of 1024 V100 GPUs for one day^62^. As this is not a viable route for us, we fine-tuned a model starting from previous checkpoints. The vocabulary used while fine-tuning a model in contrast remains the same as the underlying model which is a compromise we must accept. We used PubMedBERT as our starting point and fine-tuned it using 2.4 million materials science abstracts^19^. These abstracts were not restricted to polymers and span many different sub-domains of materials science. We restricted ourselves to abstracts because Ref. ^19^ found that a language model pre-trained on abstracts only, outperforms a language model pre-trained on abstracts as well as full text when the downstream task only involved abstracts. The computational cost incurred if abstracts, as well as full text, were to be used would also be significantly higher. The PubMedBERT model used here is itself pre-trained from scratch using the PubMed corpus (14 million abstracts from PubMed as well as full-text articles from PubMedCentral), using the BERT-base architecture. We picked PubMedBERT as our starting point as its vocabulary is specific to the biomedical domain which overlaps with materials science as material entities are frequently mentioned in biomedical papers. During fine-tuning, the model weights of PubMedBERT were loaded and training was continued using the same training objectives used to pre-train PubMedBERT but using the unlabeled text from the fine-tuning corpus as the input. The hyperparameters used during fine-tuning were identical to those used to train PubMedBERT. We used the “Transformers" library for fine-tuning PubMedBERT^63^. A similar strategy was employed in ChemBERT^23^, ClinicalBert^64^, and FinBERT^65^. We fine-tuned PubMedBERT for 3 epochs which took 90 hours on four RTX6000 16 GB GPUs to obtain MaterialsBERT.

### Material property records extraction

The trained NER model is one component of our pipeline that is used to extract material property records. Each component is explained below (Refer Fig. 1):

1. **Train NER model**: A subset of our corpus of 2.4 million papers was selected and annotated with a given ontology to train an NER model (described in the Section “NER model”). This model was used to generate entity labels for abstracts in the corpus.
2. **Pick documents with ‘poly’**: The corpus of 2.4 million abstracts was down-selected by searching for the string ‘poly’ in the abstract as a proxy for polymer-relevant documents.
3. **Run NER model**: The NER model previously trained was used for predicting entity labels on each polymer-relevant document obtained from the previous step.
4. **Abstract filtering**: As not all polymer abstracts contain material property information, the output of the NER was used as a heuristic to filter out those that do. Only abstracts with specific material entities, i.e., POLYMER, POLYMER_FAMILY, or MONOMER as well as the PROPERTY_NAME and PROPERTY_VALUE tags were allowed through this stage. This acts as a second filter to locate polymer-relevant documents.
5. **Entity extraction**: The material entities, (PROPERTY_NAME, PROPERTY_VALUE) and MATERIAL_AMOUNT entities were extracted and processed separately.
6. **Co-reference material entities**: This step was applied to co-reference all mentions of the same material entity. A common example of this is when a material is mentioned next to its abbreviation. We used the abbreviation detection system in ChemDataExtractor^3^ to find material entity abbreviation pairs. In addition, we co-referenced material entities that were within a Levenshtein distance^66^ of one. Co-referencing is a tractable problem in abstracts compared to the body of a paper as there are no long-range dependencies in the former and typically no anaphora resolution is required^67^.
7. **Normalizing polymer names**: Polymers can have several different variations in names referring to the same chemical entity. In this step, we normalized these variations to the most commonly occurring name for that particular polymer. For instance, ‘poly(ethylene)’ and ‘poly-ethylene’ occurring in different abstracts are both normalized to ‘polyethylene’. This is done using a dictionary lookup on a data set of polymer name clusters that were normalized using the workflow described in Ref. ^31^. Note that we do not normalize all polymer names but only the ones which are included in our dictionary. In practice, this includes the most commonly occurring polymers that have multiple names in the literature.
8. **Extract Property Value pairs**: The PROPERTY_NAME and PROPERTY_VALUE tag were associated by co-occurrence within a context window. The numeric value of the property was separated from the units using regular expressions and all parsed property values were converted to a standard set of units. The unit used was the most commonly reported unit for that particular property. Any standard deviation reported with the numeric value was also parsed using regular expressions.
9. **Extract Material amounts**: Entities with the MATERIAL_AMOUNT tag were extracted and the closest material entity within a context window was associated with it.
10. **Relation extraction**: In order to obtain a material property record, it is necessary to associate the material entities and the property value pair that correspond to a single record. This problem has been addressed in the literature using supervised methods^68,69^. However, the annotation process for relation labeling is time-intensive and hence we employed heuristics in this work to obtain relations between entities. To associate material entities with property value pairs, we associated the closest material entity tagged in the same sentence as the property value pair. If no such material entity was found, then all the material entities mentioned in the abstract were associated with the property value pair. This is because most commonly, an abstract mentions a major material system described in the paper and reports its measured property values. This step is reasonable in abstracts, which report this information compactly. In contrast, the body of the paper would require coreferencing the entities in text, tables, and figures to extract material property records.

## Supplementary information

Supplementary Information

## Data Availability

The journal articles used to train MaterialsBERT and to extract material property data were downloaded through licensing arrangements that Georgia Tech has with Elsevier, Wiley, Royal Society of Chemistry, American Chemical Society, Springer Nature, Taylor & Francis, and the American Institute of Physics. The pre-trained language model MaterialsBERT is available in the HuggingFace model zoo at huggingface.co/pranav-s/MaterialsBERT. The DOIs of the journal articles used to train MaterialsBERT are also provided at the aforementioned link. The data set PolymerAbstracts can be found at www.github.com/Ramprasad-Group/polymer_information_extraction. The material property data mentioned in this paper can be explored through polymerscholar.org.

## Code availability

The code used in this work can be found at www.github.com/Ramprasad-Group/polymer_information_extraction.
