# Supplementary material for: A general-purpose material property data extraction pipeline from large polymer corpora using natural language processing
Source: NPJ Comput Mater. 2023 Apr 5;9(1):52. doi: 10.1038/s41524-023-01003-w (PMC10073792; doi:10.1038/s41524-023-01003-w)
Supplement: Supplementary file 1 — Supplementary Information [file 41524_2023_1003_MOESM1_ESM.pdf]

Supplementary Materials for

A general-purpose material property data extraction pipeline from large polymer corpora using natural language processing

Pranav Shetty, Arunkumar Chitteth Rajan, Christopher Kuenneth, Sonakshi Gupta, Lakshmi Prerana Panchumarti, Lauren Holm, Chao Zhang, and Rampi Ramprasad

Corresponding author. Email: rampi.ramprasad@mse.gatech.edu

Supplementary Methods 1

Annotation guidelines

We annotated abstracts that had material property information and hence the entity types defined in our ontology. We only labeled material entities that were explicitly part of the material formulation for which a property value pair was reported in the abstract. This was done in order to encode some notion of jointly extracting entities and relationships in the model through minimal annotation effort.

1. POLYMER: All polymer material entities in a material formulation for which property values are reported in the abstract should be labeled. Abbreviations when found next to it in brackets should also be labeled (excluding brackets). This includes homopolymers, copolymers and blends. This should refer to a polymer name rather than a generic polymer family. If annotating multiple polymer entries separated by a forward slash e.g. polyethylene/polypropylene, tag each material entity on either side of the slash separately. End-functionalization of a polymer can be omitted from the labeled token.
2. POLYMER_CLASS: All polymeric material entities that refer only to polymer families (e.g. polyamide, polyimide, polybenzimidazole, etc) that occur in a material formulation for which property values are reported in the abstract should be labeled. Abbreviations when found next to it in brackets should also be tagged (excluding brackets).
3. ORGANIC_MATERIAL: Label all organic compounds that are not polymers and are used in the material formulation for which property values are reported. These could be organic molecules used as plasticizers, crosslinkers, blended with the polymer, or as grafts. These are explicitly part

of the material formulation and the extracted record should include this information.

4. MONOMER: Label all monomer repeat units for polymers for which property values are reported in the abstract. These are being annotated separately as they are not explicitly part of the material formulation of interest but are nevertheless commonly reported in abstracts. It is hence necessary to distinguish between ORGANIC additives and monomers. Look for contextual cues such as 'synthesized from', 'prepared from', or the presence of the word monomer.
5. INORGANIC_MATERIAL: Label all inorganic materials (such as SiO_2, TiO_2 etc) explicitly used in a material formulation.
6. MATERIAL_AMOUNT: Numerical quantity + unit denoting the amount of a material like additive or blend for which a property value is reported. This is typically wt % or mol.
7. PROPERTY_NAME: Name of property measured. Label properties for which a corresponding numeric value is reported in the abstract. Label the property name and abbreviation in brackets (if present) separately.
8. PROPERTY_VALUE: Numeric value + unit corresponding to a reported PROPERTY_NAME.

Supplementary Methods 2

NER Datasets used for testing BERT-based encoders

1. **ChemDNER**[1]: This is a data set of 10,000 PubMed abstracts annotated for chemical entity mentions using 7 different entity types such as ABBREVIATION, SYSTEMATIC, FORMULA, TRIVIAL, FAMILY, MULTIPLE, and IDENTIFIERS. The data set is split into 3500 abstracts for training, 3500 validation abstracts, and 3000 test abstracts.

2. **Inorganic Synthesis recipes**[2]: This is a dataset of 230 inorganic synthesis paragraphs annotated using 21 entity types that are relevant in the context of materials synthesis. 15 paragraphs each are used for validation and testing and the remaining 200 paragraphs are used for training.
3. **Inorganic Abstracts**[3]: This is a data set of 800 abstracts related to inorganic materials in which 8 different entity types related to inorganic materials are annotated namely, inorganic material (MAT), symmetry/phase label (SPL), sample descriptor (DSC), material property (PRO), material application (APL), synthesis method (SMT), and characterization method (CMT). The dataset is split as 640/80/80 for training, validation, and testing respectively.
4. **ChemRxnExtractor**[4]: This is a data set of 329 organic synthesis paragraphs in which the product of the synthesis is labeled using the BIO scheme. The data set is split into 251, 41, and 37 paragraphs in the train, validation, and test set respectively.

Supplementary Discussion 1

Performance of MaterialsBERT on PolymerAbstracts

Supplementary Table 1 Performance of an NER model using MaterialsBERT as the encoder across various entity types in the ontology used in this work on the test set of PolymerAbstracts. Values are reported in %. Total occurrences here refers to the number of occurrences of each entity type in PolymerAbstracts.

**Entity type**	**Precision**	**Recall**	**F1**	**Total occurrences**
POLYMER	75.9	83.8	79.6	7364
PROPERTY_VALUE	73.0	80.0	76.4	5800
PROPERTY_NAME	72.6	74.7	73.6	4535
ORGANIC_MATERIAL	34.7	21.0	26.2	914
MONOMER	67.1	77.6	72.0	2074
POLYMER_CLASS	41.6	52.1	46.2	1476
INORGANIC_MATERIAL	40.2	64.7	49.6	1272
MATERIAL_AMOUNT	59.1	83.9	79.6	1143

The detailed performance of MaterialsBERT on PolymerAbstracts is shown in Supplementary Table 1. There is a positive correlation between the number of occurrences of each entity type and the performance of the corresponding entity type. The ORGANIC_MATERIAL entity type has a low F1 score likely because it is difficult to distinguish between MONOMER and ORGANIC_MATERIAL entity types. The POLYMER_CLASS entity type has similarities with the POLYMER entity type which likely lowers the F1 score for this entity type as well.

Supplementary Discussion 2

Details of extracted data

The number of named entities extracted (accounting for variations in case) from the $\sim 300,000$ material property records is shown in Supplementary Table 2. Note that these material entities are not unique materials for instance, 'silica' and 'SiO_2' would be counted as separate entities but are the same material. Similarly for property names, 'T_g ' and 'glass transition temperature' are counted as separate entities, even though they correspond to the same property.

Entity type	Number of extracted entities
POLYMER	74396
MONOMER	50000
INORGANIC_MATERIAL	26114
POLYMER_CLASS	5109
ORGANIC_MATERIAL	2769
PROPERTY_NAME	33642

Supplementary Table 2 Number of entities extracted from the corpus of polymer relevant abstracts for key entity types from the ontology used in this work

The histogram of the number of records extracted for each property is shown in Figure 1. Only property entities with at least 10 material property

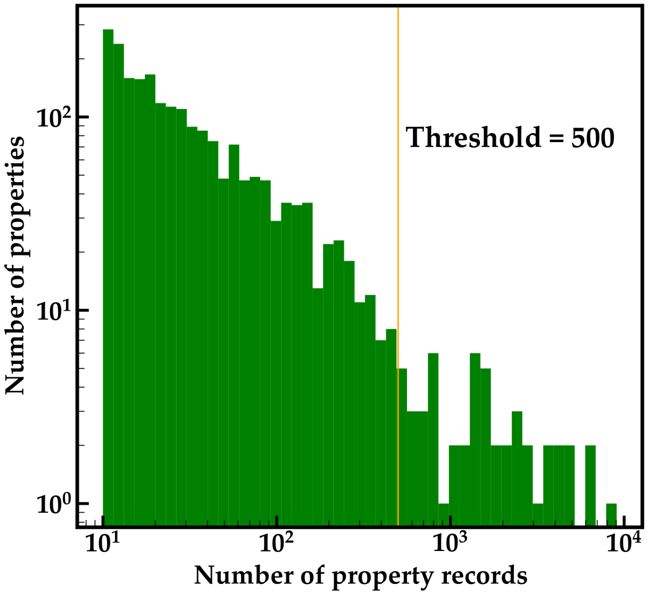

Supplementary Figure 1 Histogram of number of material property records for each property entity. The threshold represents the cut-off above which the number of material property records is reported for each property in Table 3

records are shown in this plot. Observe that the number of material property records appears to be power-law distributed with a small number of properties having a large number of records associated with them while the majority of property entities only have a small number of associated records. We threshold this plot at 500 material property records and report the number of records associated with all properties above this threshold in Table 3. Common variations in names for each property such as abbreviations (e.g. IEC for ion exchange capacity) or plural forms (e.g. activation energies) were accounted for in order to compute the number of records. These account for a total of 111279 records, i.e., 37 % of $\sim 300,000$ material property records. Device properties such as power conversion efficiency, open circuit voltage, etc listed in Table 4 of the main paper are listed in Supplementary Table 3 as well but are more in number as they are not restricted to any particular application.

Index	Property name	Number of material property records
1	Molecular weight	9053
2	Power conversion efficiency	8096
3	Glass transition temperature	6155
4	Electrical conductivity	6030
5	Ionic conductivity	4933
6	Specific capacitance	4916
7	Tensile strength	4382
8	Number average molecular weight	4096
9	Water contact angle	3932
10	Discharge capacity	3632
11	Polydispersity index	3087
12	Specific surface area	2912
13	Gravimetric power density	2816
14	Adsorption capacity	2555
15	Activation energy	2408
16	Open circuit voltage	2343
17	Band gap	2245
18	Viscosity	2084
19	Youngs modulus	1904
20	Gravimetric energy density	1765
21	Short circuit current	1699
22	Melting temperature	1615
23	Sensitivity	1602
24	Dielectric constant	1534

25	Elongation at break	1499
26	Thermal Decomposition Temperature	1479
27	Fill factor	1431
28	Thermal conductivity	1429
29	Water flux	1371
30	Density	1354
31	Current density	1337
32	Limiting Oxygen Index	1146
33	Transmittance	1135
34	Ion exchange capacity	1034
35	Porosity	1019
36	External quantum efficiency	915
37	Hole mobility	846
38	Capacity retention	843
39	Luminance	842
40	Compressive strength	814
41	Zeta potential	784
42	Sheet resistance	768
43	Lower critical solution temperature	712
44	CO_2 permeability	685
45	Resistivity	668
46	Coulombic efficiency	632
47	Crystallization temperature	605
48	Refractive index	576
49	Separation factor	543
50	Impact strength	512

51	Highest occupied molecular orbital	506

Supplementary Table 3: The number of material property records extracted for the most common properties reported in the literature. This is not a complete list of extracted properties.

Supplementary Discussion 3

Training Machine learning models using literature extracted data

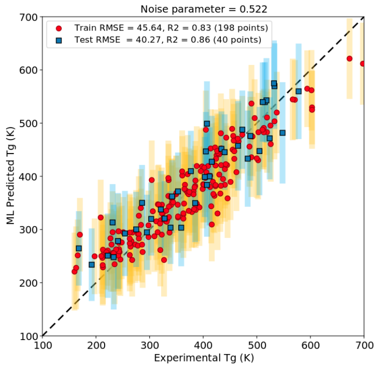

(a)

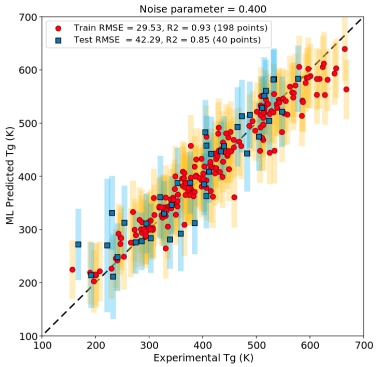

(b)

Supplementary Figure 2 Glass Transition Temperature data a) Parity plot for machine learning model trained using literature extracted glass transition temperature data, b) Parity plot for machine learning model trained using curated glass transition temperature data with the same number of points as used in a). The test set in both cases is drawn from curated data. Observe that the test RMSE in both cases is similar

In addition to the qualitative trends shown in the main paper, we also demonstrate how the data collected through our pipeline can be used as input to a machine learning model. We trained gaussian process regression models with a Matern kernel [5] to predict T_g using the material property data

extracted from our pipeline. SMILES strings [6] were used to encode the structure and endpoints of the polymer. The SMILES strings for a randomly selected subset of NLP extracted neat polymers T_g records were added manually. The SMILES string was used as the input to fingerprint the polymer and the fingerprint vector along with the literature extracted T_g value was the input to the machine learning model. The polymer SMILES string was converted to a feature vector using structural descriptors described elsewhere [7]. The feature vector consists of atomic triples, block-level features, i.e., predefined fragments such as benzene rings and ketone groups, and chain-level characteristics that are specific to a polymer such as the length of the longest side-chain, etc. This fingerprinting scheme leads to 600 fingerprint components.

Figure 2(a) shows the parity plot for a machine learning model to predict the glass transition temperature of polymers trained using data extracted from literature. For comparison, we also trained a model using the same number of data points but with polymers and their corresponding T_g values randomly sampled from a curated T_g data set reported in Ref. 28 (Figure 2(b)). The test set is the same for both cases and is taken from the curated data set. There is an 80-20 split between the train and test set in both cases. The root mean squared error (RMSE) reported here on the test set is higher than the RMSE of the best T_g models trained on larger data sets as reported in Ref. 28 ($\sim$ 20 K). However, the test RMSE of the 2 plots in Figure 2 is comparable, demonstrating that data extracted from literature can be used directly to train machine learning models of properties, without manual curation. Thus, despite being noisy, NLP extracted data in the context of organics and polymers can be used to train machine learning models which is important for NLP extracted data to be used at scale in materials informatics.

References

[1] Krallinger, M. *et al.* The chemdner corpus of chemicals and drugs and its annotation principles. *J. Cheminformatics* **7** (1), 1–17 (2015) .

[2] Mysore, S. *et al.* The materials science procedural text corpus: Annotating materials synthesis procedures with shallow semantic structures. *arXiv preprint arXiv:1905.06939* (2019) .

[3] Weston, L. *et al.* Named entity recognition and normalization applied to large-scale information extraction from the materials science literature. *J Chem Inf Model* **59** (9), 3692–3702 (2019) .

[4] Guo, J. *et al.* Automated chemical reaction extraction from scientific literature. *J Chem Inf Model* (2021) .

[5] Williams, C. K. & Rasmussen, C. E. *Gaussian processes for machine learning* Vol. 2 (MIT press Cambridge, MA, 2006).

[6] Weininger, D., Weininger, A. & Weininger, J. L. Smiles. 2. algorithm for generation of unique smiles notation. *J Chem Inf Comput Sci* **29** (2), 97–101 (1989) .

[7] Doan Tran, H. *et al.* Machine-learning predictions of polymer properties with polymer genome. *J. Appl. Phys.* **128** (17), 171104 (2020) .
